# Comprehensive Study of Sexual Reproduction in *Nicotiana tabacum* Plants Overexpressing H_2_O_2_-Producing Enzymes: Superoxide Dismutase and Choline Oxidase

**DOI:** 10.3390/plants14142103

**Published:** 2025-07-08

**Authors:** Anna Podobedova, Ekaterina N. Baranova, Alexander A. Gulevich, Inna A. Chaban, Maria Breygina

**Affiliations:** 1 Department of Plant Physiology, Biological Faculty, Lomonosov Moscow State University, Leninskiye Gory 1-12, 119991 Moscow, Russia; 17a2004@mail.ru; 2 N.V. Tsitsin Main Botanical Garden of Russian Academy of Sciences, 127276 Moscow, Russia; greenpro2007@rambler.ru; 3 All-Russia Research Institute of Agricultural Biotechnology, Timiryazevskaya St. 42, 127550 Moscow, Russia; a_gulevich@mail.ru

**Keywords:** SOD, ROS, plant reproduction, glycine-betaine, genetic engineering

## Abstract

Transgenic tobacco plants with additional enzymes producing hydrogen peroxide (H_2_O_2_)—superoxide dismutase from *Arabisopsis thaliana* and choline oxidase from *Arthrobacter globiformis*—have increased resistance to stress factors, which was demonstrated previously, but their reproductive potential has not been studied to date. Superoxide dismutase converts superoxide radical into H_2_O_2_, and choline oxidase catalyzes the oxidation reaction of choline to form betaine aldehyde, which is subsequently converted into glycine betaine and H_2_O_2_. We found that the addition of both exogenous genes stimulated growth of the floral organs: petals, styles, and stamens. However, the reproductive potential of the transgenic plants was different. Thus, the introduction of the superoxide dismutase gene *FeSOD* significantly increased pollen germination in vitro, in vivo, the size of fruits, and the number of seeds. At the same time, the insertion of the *CodA* gene resulted in the production of abnormal pollen with low germination in vitro. The female reproductive potential system in these plants was not affected. Thus, shifting the ROS balance towards hydrogen peroxide not only increases tobacco stress resistance but also stimulates reproductive success. Glycine betaine production disrupts pollen formation, although such plants show increased resistance to osmotic stress.

## 1. Introduction

The use of a genetic engineering approach to increase the resistance of agricultural plants to unfavorable environmental factors is a relevant method [1], which, however, has potential side effects [2]. Thus, by introducing some additional genes important for photosynthetic tissues (including those coding proteins with plastid localization), one cannot take into account all the effects of this gene in various tissues and organs of the whole plant [3]. In such cases, it is important to pay attention to the sexual reproduction of plants, on which genetic engineering can have an unpredictable effect. For example, mutations in some of the genes involved in epigenetic processes during stress were shown to cause changes in flowering [4]: late flowering of the freezing-sensitive Arabidopsis mutant *hos15* was shown to result from deacetylation of the f lowering genes SOC and FT [5]. The flowering repressor FLC (a MADS-box protein) is epigenetically repressed during vernalization, allowing the acquisition of the competence to flower after exposure to prolonged low temperatures [4]. This process was shown to involve numerous proteins with potential to alter chromatin remodeling including VIN3, FCA, and FPA [6]. In addition, it was recently shown that VIN3 is also responsive to hypoxic conditions, suggesting that other abiotic stresses might affect flowering time via modifications of this pathway [7].

One of the genes that increases plant resistance to adverse factors is choline oxidase (Cod) [8]. Engineering plants with additional choline oxidase activity has shown success in increasing plant adaptability, growth, and photosynthetic characteristics [9,10,11]. This enzyme is interesting because it leads to the synthesis of two products: betaine aldehyde, which is subsequently converted into glycine betaine, and H_2_O_2_ [10]. Glycine betaine is a protector that helps cells by acting as an osmolyte, maintaining the water balance and stabilizing macromolecules during cell dehydration and high salt concentrations, i.e., acting as a molecular chaperone [12,13]. The accumulation of glycine betaine helps plants stabilize the osmotic potential, which is especially important for resistance to salinity [14,15]. At the same time, hydrogen peroxide in an important physiological signal [16,17], and it is possible that both products of the COD-driven reaction might activate stress response pathways and prepare transgenic plants to mitigate stress. In rice, microarray-based transcriptome analysis unraveled altered expression of many genes involved in stress responses, signal transduction, gene regulation, hormone signaling, and cellular metabolism. Out of 165 differently expressed genes, at least 50 genes are known to be involved in plant stress response. Exogenous application of both H_2_O_2_ or glycine betaine to wild-type plants also induces the transcription of such genes [9].

If the plant’s resistance is at least partly due to the production of hydrogen peroxide, it is possible to test the enzyme that synthesizes it as the main reaction product, for example, superoxide dismutase. Historically, SOD has been considered to have an antioxidant activity that is induced in response to stress with the sole purpose of reducing superoxide, yet these enzymes also produce H_2_O_2_ as a signaling molecule [18]. The introduction of an additional gene for this enzyme has also been used in many cases to increase plant resistance to stress. Overexpression of various superoxide dismutase genes has proven to be a very interesting and promising strategy. Due to the mass production of H_2_O_2_, other antioxidant defense enzymes are activated, which leads to an increased resistance under adverse stress conditions such as salt, drought, or cold but does not improve plant growth under optimal conditions [19,20,21].

Hydrogen peroxide is a central participant in signaling processes associated with both stress and sexual reproduction in plants [22,23]. Earlier studies found that low H_2_O_2_ concentrations (100 μM) stimulated tobacco pollen germination in vitro, while high concentrations (2 mM and higher) inhibited it [24]. At the same time, staining of the pistils showed the presence of ROS on them [25,26]. Later we found that hydrogen peroxide is massively produced on the tobacco stigma, with SOD involved in the process [27]. According to inhibitor analysis, two SOD isoforms are active in tobacco stigma, one of which is FeSOD localized in plastids [28]. Blocking one of the SOD isoforms on the stigma inhibited pollen tube growth in vivo and severely reduced seed production [27]. The next step was to increase peroxide production within physiological limits in order to stimulate pollen germination. To do this, we will compare the results of pollination of transgenic plants with two enzymes that catalyze the production of H_2_O_2_. Transgenic *Nicotiana tabacum* plants expressing *Arabidopsis* superoxide dismutase (*AtFeSOD*) [29] or *Arthrobacter globiformis* choline oxidase (*AgCodA*) [30,31] have proven themselves to be more effective in stressful conditions than wild-type plants. However, since pollen germination on the stigma is a process with complex regulation [22], and many enzymes play a role in determining the redox status, including peroxidases [32,33], one should interpret the results with caution.

## 2. Results

### 2.1. FeSOD and CodA Influence Organ Lenght

**Figure 1:**
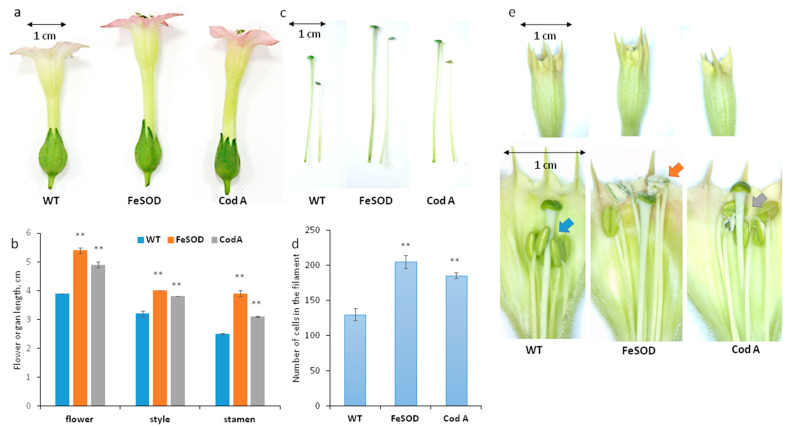
The length of flowers (**a**,**b**), elongated parts of the flower (**b**,**c**), number of cells (**d**), and appearance of pre-mature flowers (**e**) in tobacco plants of 3 genotypes (wild type, *FeSOD*, and *CodA*): characteristic photos (**a**,**c**,**e**) and diagrams with average values (**b**); (**a**–**e**) in *FeSOD* and *CodA*, all the flower organs are longer; (**d**) the number of cells in one row in the stamen filament is higher in *FeSOD* and *CodA* compared to the wild type; (**e**) as a result of uneven elongation, the relative position of anthers and stigma changes in transgenic plants: in the wild type, the stamens do not reach the stigma; in *SOD* plants, they are at the same level; in *CodA*, they are close; in *FeSOD*, the anthers open at an earlier stage (flowers before preparation can be seen in the upper part); Red arrow—very highly elongated stamens; blue—normal elongation of stamens of the control flower; gray arrow—stimulation of stamen elongation. ** *p* < 0.01.

Since previous studies focused on the resistance of transgenic plants to stress factors, and we studied their reproductive potential, the plants were grown under optimal conditions. Expression of inserted genes (*AgCodA* and *AtFeSOD*) was previously tested in vegetative organs [29], but it is also known that in tobacco genes under the 35S promoter are expressed in virtually all tissues and organs except gametophytes with high expression in anthers, filaments, petals, and styles [34]. It turned out that plants with an additional *FeSOD* and *CodA* genes have longer flowers (Figure 1a,b) and elongated floral organs: stamens and style (Figure 1b,c).

The length of the flower organs changed unevenly: the stamen filaments in transgenic plants are longer relative to the style than in wild-type plants (Figure 1e). In control plants, the stamens do not reach the stigma. In *FeSOD* transgenic plants, the stamens and pistils are the same length. In *CodA* transgenes, an intermediate pattern is observed (Figure 1e): their stamens are closer to the stigma than in wild-type plants, but not at the same height. As an observation, we also noted that in *FeSOD* transgenic plants, anther dehiscence occurred at an earlier stage of flower development than in the wild type: on the eve of the stage of full fertility, corresponding to a wet stigma and an open corolla, the anthers in this genotype were already fully open while in the wild type they were closed (Figure 1e, anthers marked by arrows).

The first hypothesis to explain the increase in the floral organs’ length was the increased growth of cells. We tested this hypothesis by measuring the length of cells in the filament and style of wild-type and transgenic plants in the zone 1 cm from the anther or ovary. It turned out that the length of the cells did not differ significantly (Table 1). We tested this hypothesis by counting all cells in the filament in one row from the base of the filament to the base of the anther. The number of cells was significantly different between the wild type and both transgenes (Figure 1d). Apparently, the stimulation is due to the increased cell number, not cell expansion. And, judging by the fact that this effect was manifested in both transgenic lines, it was a consequence of excess hydrogen peroxide.

### 2.2. FeSOD Enhances Male Fertility, Whereas CodA Reduces It

An important indicator of the reproductive potential of transgenic plants is pollen quality, which can be assessed visually using scanning electron microscopy, by assessing pollen viability, and also by testing its germination in vitro. All these observations showed that the introduced genes act differently on pollen maturation. Plants with additional *FeSOD* have excellent-quality pollen and fewer aborted pollen grains than the wild type, and plants with *CodA* gene have low-quality pollen, whose viability and germination are critically low.

**Figure 2:**
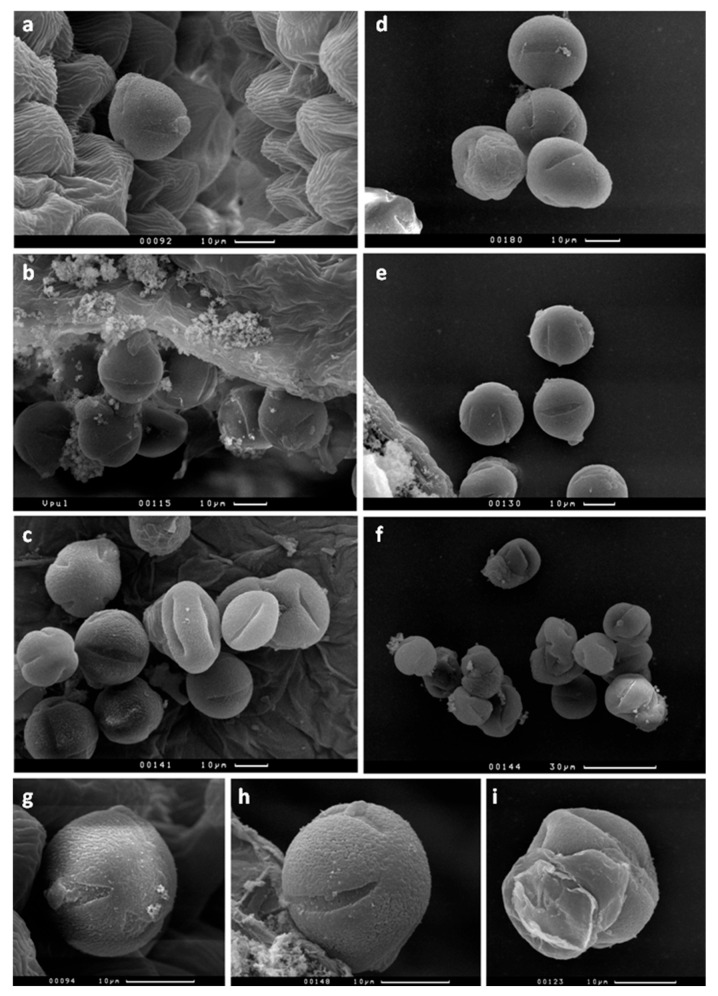
SEM images of pollen in the anther at a late stage of maturation (**a**–**c**) and mature at the presentation phase (**d**–**f**), at larger magnification (**g**–**i**) for plants of 3 genotypes: wild type (**a**,**d**,**g**), *FeSOD* (**b**,**e**,**h**), and *CodA* (**c**,**f**,**i**). Anthers in *FeSOD* transgenic plants mature earlier than in the wild type, relative to other parts of the flower; therefore, when the anther in SOD has already completely dried out and its wall has opened (**b**,**h**), in control plants and *CodA* transgenic line the anther walls are still represented by living cells (**a**,**c**,**g**). Normal pollen grains predominate in the first two genotypes (**a**–**e**,**g**,**h**), while *CodA* transgene has a wide range of abnormal grains that will not be able to perform their functions (**c**,**f**,**i**).

**Figure 3:**
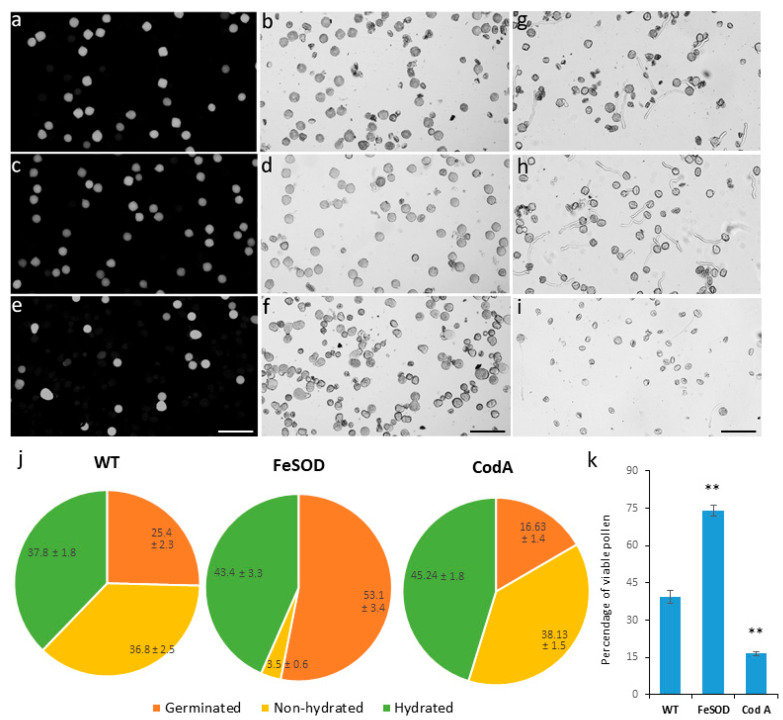
Pollen viability and in vitro germination for tobacco plants of 3 genotypes. (**a**–**f**,**k**) Viability of mature pollen, which was assessed using the standard FCR test (FDA fluorescence and corresponding pollen grains in a bright field): (**a**,**b**) wild type, (**c**,**d**) *FeSOD*, (**e**,**f**) *CodA*, (**k**) corresponding quantitative data; (**g**–**i**) pollen germination in vitro: characteristic bright field images ((**g**) wild type, (**h**) *FeSOD*, (**i**) *CodA*) and ratios of pollen grain categories after 1 h of incubation (**j**). Scale bar—100 µm. In the wild-type and *SOD* transgenic pollen suspensions, viability is good (**a**,**c**,**k**); there are few abnormal grains, while *CodA* transgenic plants have a large number of defective pollen (**e**,**k**). Pollen germination in *SOD* plants significantly exceeds that of the wild type and *CodA* (**g**–**j**). ** The *p* value is less than 0.01.

A study of mature pollen using SEM at the stage of presentation from the anther showed that in the two genotypes—wild type and *FeSOD—*the pollen wall has normal appearance, i.e., the grain has a standard shape and four apertures (Figure 2a–e,g,h), while *CodA* transgenic plants have many abnormal grains with a damaged surface (Figure 2c,f,i). These differences are also reflected in the viability of mature pollen, assessed by a standard fluorochromatic (FCR) test (Figure 3k). Thus, pollen from wild-type and *SOD*-transgenic plants has normal viability, as can be seen in Figure 3a–d. These images also show a smaller amount of defective pollen in *FeSOD* transgenic line compared to the wild type. Pollen from plants with the *CodA* gene has very low pollen viability (Figure 3e,f,k).

Pollen germination in optimal in vitro conditions followed the same pattern: the percent of germinated grains in 60 min for wild-type plants was 25.4, while for *FeSOD* transgenic plants it was twice as much—53.1% (Figure 3g,h). In *CodA* transgenes, only 16.6% germinated (Figure 3i). Grains that did not form pollen tubes were divided into two categories: (1) ungerminated but hydrated and (2) unhydrated. The former can potentially germinate in case of prolongated incubation, while the latter are aborted non-viable pollen grains. The ratio of not only germinated-to-ungerminated pollen grains differs in the wild-type and *SOD* transgenes but also between the two categories of ungerminated pollen grains: in *FeSOD*-transgenic pollen the percentage of non-hydrated grains is an order of magnitude lower (Figure 3j). The difference in germination between the wild type and *CodA* transgenic line is also significant (*p* < 0.01), but it is due to hydrated ungerminated grains: *CodA* has significantly more of them (*p* < 0.01) (Figure 3j).

Thus, male fertility of *FeSOD* transgenic plants is significantly increased, while *CodA* has the opposite effect, reducing the quality and germination of pollen.

**Figure 4:**
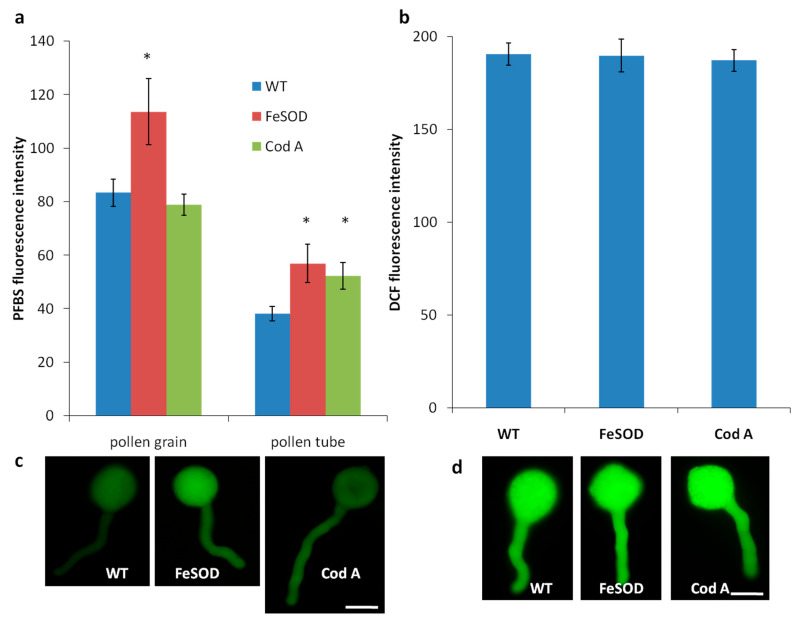
Levels of hydrogen peroxide (**a**,**c**) and total ROS (**b**,**d**) in pollen of the three tobacco genotypes germinating in vitro assessed by PFBS (**a**,**c**) and DCFH-DA (**b**,**d**) staining. PFBS fluorescence intensity was measured in both grains and tubes (**a**); DCF—in pollen tubes (**b**), as in pollen grains it was too unstable. DCFH-DA staining showed no differences between genotypes; PFBS staining showed higher peroxide levels in pollen grains and tubes of *FeSOD*-transformed plants and in pollen tubes of *CodA*-transformed plants. * *p* < 0.05. Scale bar—20 µm.

We tested whether the increased pollen germination of the *FeSOD* genotype is associated with the amount of hydrogen peroxide and total ROS in pollen grains. Using staining of germinating pollen grains with specific and non-specific dyes, we found that pollen tubes of the three genotypes do not differ in the total ROS level (Figure 4b), while the level of hydrogen peroxide in pollen grains and tubes of the *FeSOD* genotype is significantly higher (Figure 4a).

### 2.3. CodA Does Not Increase Pollination Success, While FeSOD Does

Pollen germination speed and pollination success in transgenic plants were tested using standard pollen from plants of the same species, the Petit Havana variety, which is characterized by a high percentage of viable pollen grains. The stigmas of all genotypes were pollinated with a standard pollen sample. Then the flowers were either fixed after 3 or 5 h, or they were left until the fruits ripened.

**Figure 5:**
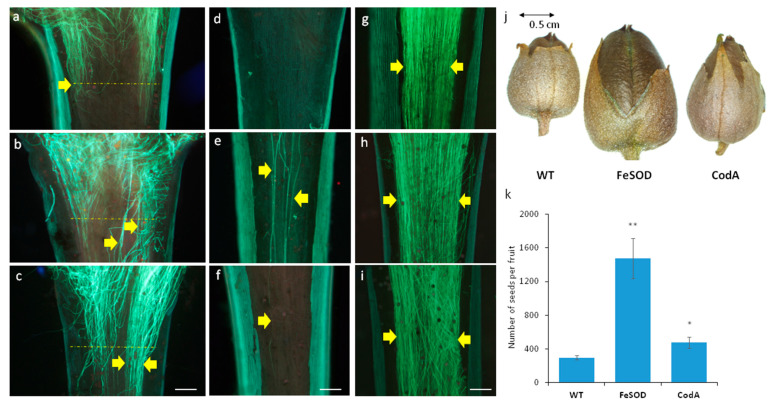
Pollen germination in vivo and seed productivity in tobacco plants of 3 genotypes: (**a**–**i**) sections of styles 3 h (**a**–**f**) and 5 h after pollination (**g**–**i**) in the upper part bordering the stigma (**a**–**c**) and in the middle part (**d**–**i**) for wild type (**a**,**d**,**g**), *FeSOD* (**b**,**e**,**h**), and *CodA* (**c**,**f**,**i**); (**j**) characteristic appearance of the capsule; (**k**) average seed set for 3 genotypes, * *p* < 0.05, ** *p* < 0.01. Scale bar in (**a**–**i**)—200 µm. In the styles of all genotypes, 3 h after pollination, pollen tubes (marked by yellow arrows) grow into the style but in control and *CodA* transgenic plants, they do not reach its middle (**d**,**f**), while in *FeSOD* plants the tubes already grow in the middle part of the style (**e**). Yellow dashed lines indicate the growth front of pollen tubes. Acceleration of pollen tube growth also leads to an increase in seed productivity: the number of seeds and the size of the fruit increase significantly in transgenic plants.

When evaluating germination in vivo, two reference points were considered: the beginning of the style (Figure 5a–c, yellow line) and the middle of the style (Figure 5d–i). This allowed us to estimate the growth rate of pollen tubes. In wild-type plants, after 3 h, pollen germinated on the stigma and began to enter the style (Figure 5a), but the tubes did not grow down to the middle of the style (Figure 5d). However, after 5 h, the tubes passed through the middle of the style (Figure 5g) and reached the ovary. In plants with an additional *FeSOD* gene, after 3 h tubes were already present both at the border of the stigma and style and in the middle of the style (Figure 5b,e). After 5 h, there were more tubes (Figure 5h). In plants with *CodA* gene, the pattern did not differ significantly from the wild type (Figure 5c,f,i).

Similar trends were observed after fertilization. We visually assessed the capsules (fruits) (Figure 5j) and quantitatively estimated the efficiency of fertilization by the number of seeds in the capsule. In the capsules of wild-type and *CodA* transgenic plants, the number of seeds was 300 and 450, respectively, while in the capsules of *FeSOD* transgenes there was 3–4 times more seeds (Figure 5k), and the fruits were visually much larger (Figure 5j). Thus, the reproductive advantage of *FeSOD* transgenic plants in the female sphere is obvious, while for *CodA* only a weak effect was observed.

## 3. Discussion

Hydrogen peroxide is a significant regulator of sexual reproduction in tobacco. It accumulates in stigma exudate [28]. In plant tissues, the main pathway for generating hydrogen peroxide is the SOD-mediated dismutation of O^•^_2_^−^, which, in turn, is formed both on the plasma membrane and in various organelles, including plastids [35]. There are potential sources of apoplastic origin of hydrogen peroxide including peroxidases, amine oxidases, and oxalate oxidases [36]. H_2_O_2_ is a product of reactions mediated by oxidases, such as diamine oxidases and polyamine oxidases, which are involved in polyamine degradation [37]. Processes like photorespiration and fatty acid oxidation generate H_2_O_2_ in peroxisomes and glyoxisomes [35]. In the present study two pathways for generating peroxide were used, one of which is natural to plants (via SOD), while the other occurs naturally in animals and bacteria (via choline oxidase) but is actively introduced into plants through genetic engineering [9,11,20,38]. As for increasing the resistance of plants to stress, both introduced genes showed excellent results described previously [29,30], but their impact on the reproductive sphere turned out to be completely different.

SOD is one of the key enzymes in pollen–stigma interactions for plants with wet stigma, including the agriculturally important representatives of the Solanaceae family [39]. The results demonstrate that additional SOD stimulates in vivo pollen germination in tobacco transgenic plants: pollen tubes grow faster into the style, and fruits are larger and contain more seeds than in the wild type (Figure 5). This is consistent with previous data that SOD activity in tobacco stigma is maximal at the fertile stage [28], and inhibition of a single SOD isoenzyme dramatically reduces both the number of tubes grown into the style and seed set [27].

Completely new results were obtained when studying the pollen quality and in vitro germination in *FeSOD*-transformed plants (Figure 2 and Figure 3): they produced high quality pollen with increased germination compared to the wild type. This confirms the data obtained earlier on control pollen: addition of active SOD enzyme to an in vitro suspension stimulated pollen germination [24], although not as significantly as the transformation performed in this study. During germination, tobacco pollen grains produce ROS; low concentrations of ascorbic acid reduced their level and stimulated germination; further reduction in ROS concentration, however, had a negative effect [40]. We also found an increase in the level of hydrogen peroxide in the pollen of SOD-transformed plants at a constant amount of total ROS (Figure 4). This means that it is the high proportion of hydrogen peroxide among other endogenous ROS that positively affects pollen germination. The dynamics, balance, and interaction of ROS and redox balance enzymes participating in the reproductive processes are regulated at a subtle level [22,41].

Thus, enhancing SOD activity through genetic engineering produces plants with improved reproductive potential: both pollen and stigmas of such plants cope more successfully with their functions. Apparently, this is due to a shift in the ROS balance from superoxide radical towards hydrogen peroxide (Figure 4) and, possibly, further ROS elimination by other redox enzymes: thus, the activity of ascorbate peroxidase was increased in the leaves of *FeSOD* transgenic plants [29]. It is known that peroxidases actively work on the stigma [33] and in tobacco are responsible for the elimination of hydrogen peroxide, since catalase is inactive in this tissue [28].

H_2_O_2_ production in a reaction involving another enzyme, choline oxidase, gave an effect similar on increasing the length of flowers and their individual parts (Figure 1). Apparently, this effect is due to the presence of peroxide and is not interfered with by the second reaction product, glycine betaine. Other positive effects on the reproduction were weak in *CodA* transgenes. This supports the idea that these plants grow better than controls under stressful conditions, but under normal conditions they lag behind and have defects: as previously reported, *CodA*-plants had a delayed transition to flowering [31]. A detailed study of their sexual reproduction capabilities showed that they have severely impaired male fertility: low pollen quality and impaired germination (Figure 2 and Figure 3). Since the effect in *SOD*-transgenic plants is the opposite, it can be concluded that the disturbances are caused not by H_2_O_2_ but by glycine betaine, which, as previously shown for the vegetative organs of *CodA*-transgenic line, accumulates in the vegetative plant tissues [31]. The simplest explanation behind low-quality pollen is the osmotic activity of glycine betaine, which in a stressful situation allows better tolerance to drought [3,15,42] but, presumably, disrupts pollen maturation for which dehydration is necessary. During development, pollen grains absorb water and increase in volume, then loose water reaching a minimum content when fully mature [43]. Pollen desiccation induces a metabolically dormant state that enables survival of environmental stresses experienced during pollen dispersal [44]. Fine-tuning of this process is crucial for maintaining the viability of pollen and is species-dependent [45]. Pollen grains that do not undergo complete dehydration are called partially hydrated, and such pollen is extremely short-living, since it does not have mechanisms for preserving moisture in dry ambient air [43,46]. Tobacco is characterized by relatively deep dehydration of pollen and long-term preservation of the germination ability [46]. In *CodA* transgenic plants dehydration may be disrupted by high concentrations of glycine betaine, which explains multiple abnormal pollen grains observed by SEM (Figure 2). At the same time, reproductive success from the female side does not differ or is slightly better than in the wild type, which can be explained by the small generation of peroxide and the lack of influence of glycine betaine on the functions of the pistil (Figure 5). Thus, for the reproductive potential of tobacco, choline oxidase is of no interest, and it is preferable to propagate such plants vegetatively or pollinate with wild type pollen.

In this study, we did not test the expression levels of *SOD* and *CodA* genes in different flower organs. We cannot exclude that the differences in effects of the two genes are related to the expression level, but this seems unlikely since both were inserted under the same promoter (35S), which is constitutive and whose expression has been well studied in tobacco [47]. In mature plants, genes under this promoter are actively expressed in flowers, especially in petals, the basal part of a pistil, stamen filaments, and anthers [34]. More likely, the introduction of genes for additional H_2_O_2_-producing enzymes leads to changes in the expression and/or activity of other redox metabolism proteins, such as peroxidases. This could be the subject of future research.

## 4. Materials and Methods

### 4.1. Plant Cultivation and Pollination In Vivo

Tobacco plants (*Nicotiana tabacum* L.) cv. Samsun, transformed as described earlier [31] using *Agrobacterium* strain AGL0 with a genetic construct containing the choline oxidase synthesis gene from *Arthrobacter globiformis* (*codA*) or the Fe-containing superoxide dismutase (*FeSOD*) gene from *Arabidopsis thaliana* L. under the control of a constitutive CaMV 35S promoter, were used in this study. The presence of transgene in plants has been proven by PCR. The expression of the *CodA* gene in transgenic plants was shown by RT-PCR earlier [31]. The synthesized SOD was targeted into the plastid due to a fused signal sequence from the pea ribulose bisphosphate carboxylase/oxygenase gene [19].

In vitro, plantlets were propagated by cuttings on the modified Murashige–Skoog basal medium (Sigma-Aldrich, Burlington, MA, USA) (1/2 macrosalts, full composition of microsalts and iron chelate without plant growth regulators, supplemented with 0.7% sucrose and 0.7% agar-agar) and maintained in the phytotron chamber with a 16/8 h (day/night) photoperiod at an illumination intensity of 250 µM m^−2^ s^−1^ and temperatures of 24–25/19–20 °C (day/night). Before adapting to the soil conditions (commercial universal soil mixed with perlite 3:1), in vitro, plantlets were soaked in sterile distilled water and transferred to the soil in one day.

Mature plants of *Nicotiana tabacum* L. of three genotypes based on var. Samsun SR were grown in a climatic chamber in controlled conditions (25 °C, 16 h light) in vermiculite. The plants were watered with salt solutions [48]. The plants of *N. tabacum* var Petit Havana were grown in the same chamber to produce pollen. Pollination material: anthers were removed from Petit Havana plants before flower opening and dried at 25 °C for three days. Mature pollen was collected and preserved at −20°. For controlled pollination standard sample of defrosted pollen (1 mg) was added to a receptive pistil and evenly distributed over with a spatula. Pollen tube growth into the style was evaluated after 3 and 5 h, seed set—after 3–4 weeks, when the fruits were completely dry.

### 4.2. Evaluation of Pollen Germination In Vivo and Fertilization Success (Seed Set)

In vivo germination rates were assessed on longitudinal sections of styles. Styles were fixated in 70% ethanol for 30 min, cut lengthwise, and placed into 70% ethanol for 1 more day. The pistils were placed cut-side up on a glass slide, and 8 M KOH was added, covered with a cover glass and left for 40 min. The alkali was drawn off using filter paper, and then stained (0.1% (*w*/*v*) aniline blue in 108 mM K_3_PO_4_) for 1 day to visualize pollen tube callose [49].

The seed set was evaluated by weighing the entire mass of seeds removed from the capsule. Calibration carried out on numerous samples allowed us to establish a linear correlation between the total weight and the number of seeds. Seeds of plants of different genotypes are identical in weight and size (1 seed = 64 µg).

### 4.3. Pollen Germination In Vitro

Pollen germination efficiency was assessed after 1 h of cultivation at 25 °C in standard medium containing 0.3 M sucrose, 1.6 mM H_3_BO_3_, 3 mM Ca(NO_3_)_2_, 0.8 mM MgSO_4_, and 1 mM KNO_3_ in 25 mM MES-Tris buffer, pH 5.8. Before cultivation pollen was pre-hydrated in a humid atmosphere for 2 h. Germinated pollen was fixed with 2% paraformaldehyde in 50 mM Na-phosphate buffer, pH 7.4, for minimum 30 min at 4 °C. Between 500 and 900 pollen grains were counted for each sample.

### 4.4. Organ Measurements

The length of organs was measured in different individuals of mature plants. Length measurements: for flowers—from the receptacle to the upper protruding part of the corolla, for stamens—from the place where they attach to the corolla to the upper edge of the anthers, for pistils—from the upper edge of the ovary to the upper edge of the stigma.

### 4.5. ROS Measurements

To determine ROS content, pollen grains were cultured in vitro in the same medium as for counting germination. After 40 min, the dye was added to the medium to the final concentration. To assess total ROS, we used DCFH-DA (Sigma-Aldrich, Burlington, MA, USA) (20 µM). To assess the level of H_2_O_2_, the cells were stained with pentafluorobenzenesulfonyl fluorescein (PFBSF) [50] (Santa Cruz Biotechnology, Santa Cruz, CA, USA) (10 µM). Staining was performed at 25 °C for 15 min.

### 4.6. Cell Length Measurements and Counting Cells

The length of the cells and their number were determined from photographs taken with a fluorescent microscope. The cells were stained with DAPI (1 mg/L in alcohol) to make it easier to count the nuclei. The cell membranes have autofluorescence, so the length of the cells could be determined without difficulty. Examples of photographs used for counting can be seen in the Supplementary Materials.

### 4.7. Fluorescence Microscopy

Quantitative and qualitive fluorescence microscopy was performed with a widefield fluorescence microscope Axioplan 2 imaging MOT (Carl-Zeiss-Stiftung, Oberkochen, Germany) equipped with an ADF Pro 08 digital camera and a mercury lamp. For DCFH-DA and PFBSF, a FITC filter set was used. Photos of cells stained with DAPI or with autofluorescence were made with a DAPI filter set.

### 4.8. Statistical Analysis

Experiments were performed in four to seven independent replications. Results in the text and figures, except the original pictures, are presented as means ± standard errors. Normal distribution was checked by the Anderson–Darling test. Significant difference was evaluated according to ANOVA for normally distributed samples. For those samples that did not pass the normality test, the Kruskal–Wallis test was applied.

## Figures and Tables

**Table 1:** Cell length in elongated organs of tobacco flowers, µm.

Genotype	Filament	Style
WT	219.5 ± 6.8	261 ± 7.2
FeSOD	214.5 ± 5.8	271.7 ± 7.3
CodA	223.6 ± 4.7	268.3 ± 7

## Data Availability

Data are contained within the article and Supplementary Materials.

## Supplementary Materials

The following supporting information can be downloaded at: https://www.mdpi.com/article/10.3390/plants14142103/s1, Figure S1: DAPI stained filaments; Figure S2: ROS detection on stigma; Figure S3: Length of cells in filaments; Figure S4: Length of cells in style.
