# Supplementary material for: Comprehensive Study of Sexual Reproduction in Nicotiana tabacum Plants Overexpressing H2O2-Producing Enzymes: Superoxide Dismutase and Choline Oxidase
Source: Plants (Basel). 2025 Jul 8;14(14):2103. doi: 10.3390/plants14142103 (PMC12299658; doi:10.3390/plants14142103)

Supplementary Materials 1

Figure S1. DAPI stained filaments.

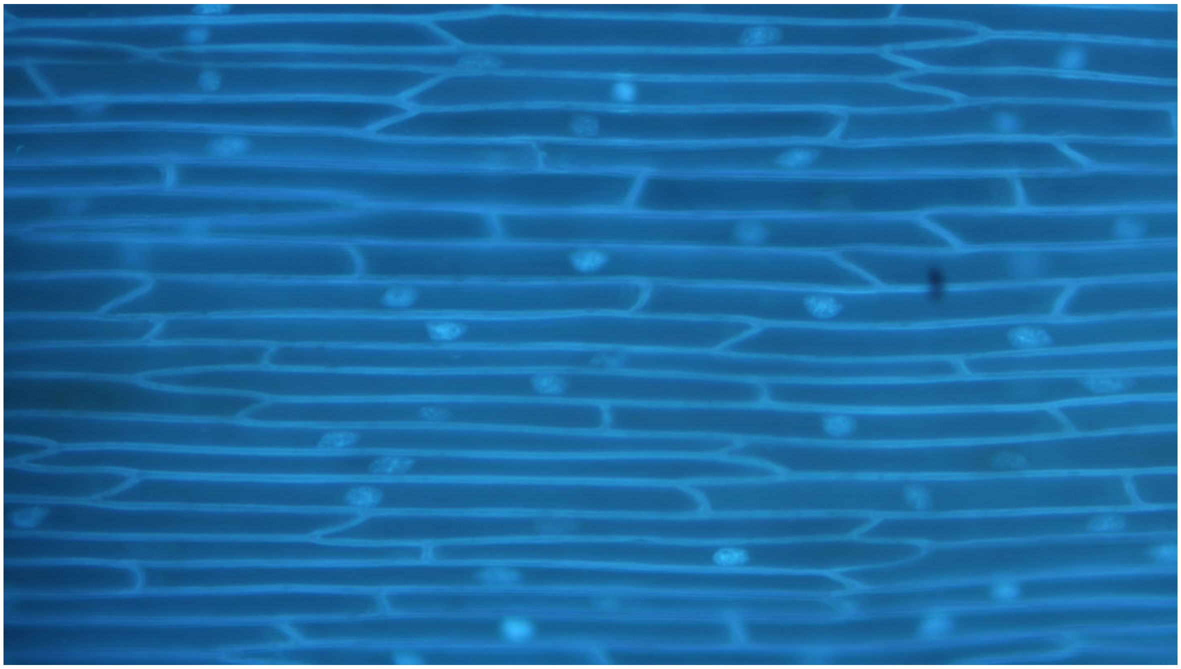

Figure S2. ROS detection on stigma.

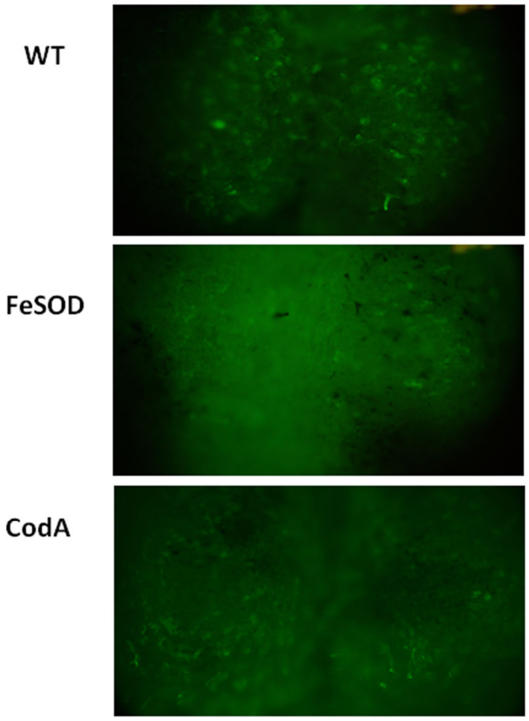

Staining: DCFH deesterified 20 μM 15 min
Deesterification: DCFH-DA + 10 mM NaOH 1 h 25◦C

Figure S3. Length of cells in filaments

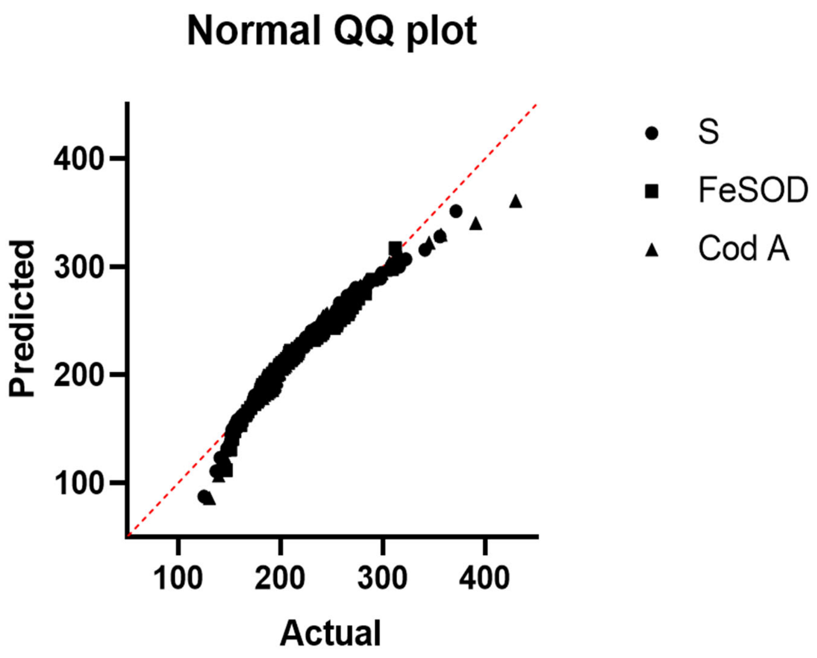

Figure S4. Length of cells in style

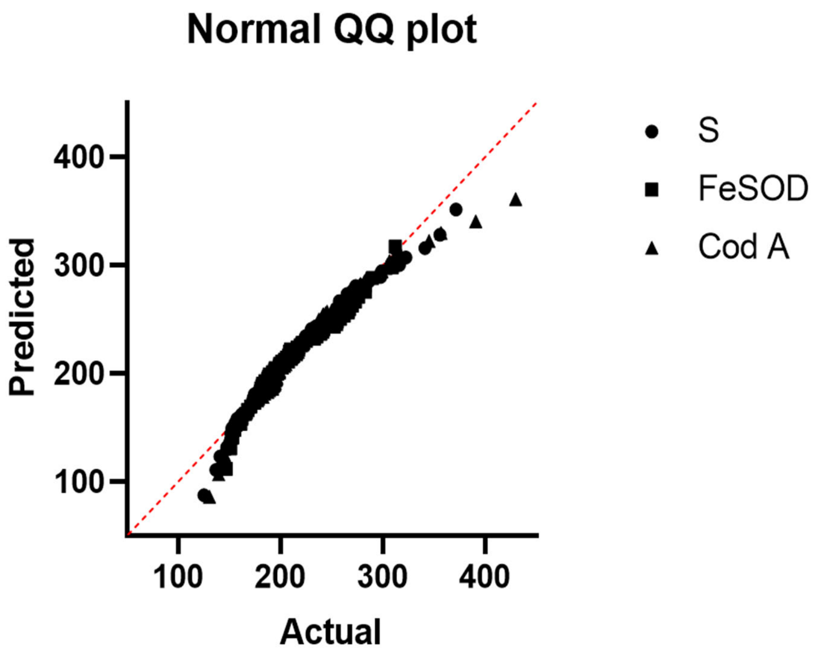

Supplement: Supplementary file 1 [file plants-14-02103-s001.zip › plants-3665833-supplementary.pdf]
